# Supplementary material for: The antimicrobial fibupeptide lugdunin forms water-filled channel structures in lipid membranes
Source: Nat Commun. 2024 Apr 25;15:3521. doi: 10.1038/s41467-024-47803-6 (PMC11045845; doi:10.1038/s41467-024-47803-6)
Supplement: Supplementary file 1 — Supplementary Information [file 41467_2024_47803_MOESM1_ESM.pdf]

Supporting Information

Unveiling the mode of action: The antimicrobial fibupeptide lugdunin forms water-filled channel structures in lipid membranes

Dominik Ruppelt, Marius F. W. Trollmann, Taulant Dema, Sebastian N. Wirtz, Hendrik Flegel, Sophia Mönnikes, Stephanie Grond, Rainer A. Böckmann,* Claudia Steinem*

Chromatograms for synthesis of methylated peptides 3-5 2
Fragment ion analysis by LC-HRMS for *N*-methylated lugdunin analogues 3-5 3
NMR spectra of *N*-methylated lugdunin analogues 3-5 4
Detailed experimental setup for orientational IR studies 7
Molecular dynamics simulations of lugdunin 7
Partitioning of lugdunin into different lipid compositions 13
Molecular orientation of lipid multi-bilayers 16
IR spectra of lugdunin for different peptide-to-lipid ratios 17
Lucigenin quenching assay 17
Setup and analysis of molecular dynamics simulations of channels composed of lugdunin peptides 18
Single-channel properties of lugdunin nanotubes 21
Supplementary Movies 22

Chromatograms for synthesis of methylated peptides 3-5

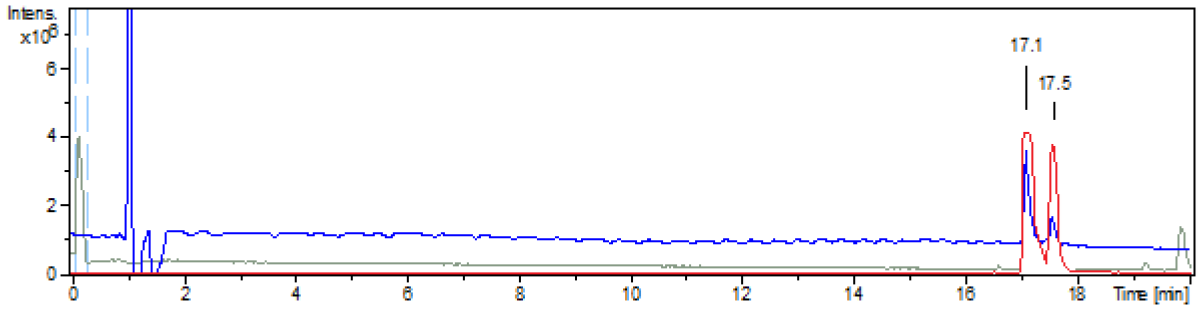

Supplementary Fig. 1 | HPLC-UV-MS chromatogram of **3** (Nucleoshell® EC RP-C18 column, 150 × 2 mm, 2.7 µm, flow rate: 0.3 mL min^{-1} with a linear gradient from 90:10 (0.1% FA in H_2O:0.06% FA in MeOH, *v*:*v*) to 0:100 (0.1% FA in H_2O:0.06% FA in MeOH, *v*:*v*) over 20 min. Monitored at λ = 220 nm. The BPC (base peak chromatogram) is shown in gray and the EIC of the *N*-methylated peptide **3** ($C_{47}H_{64}N_9O_6S$) with $[M+H]^+$, *m/z* 884.485 ± 0.005 for $C_{47}H_{65}N_9O_6S$ is shown in red.

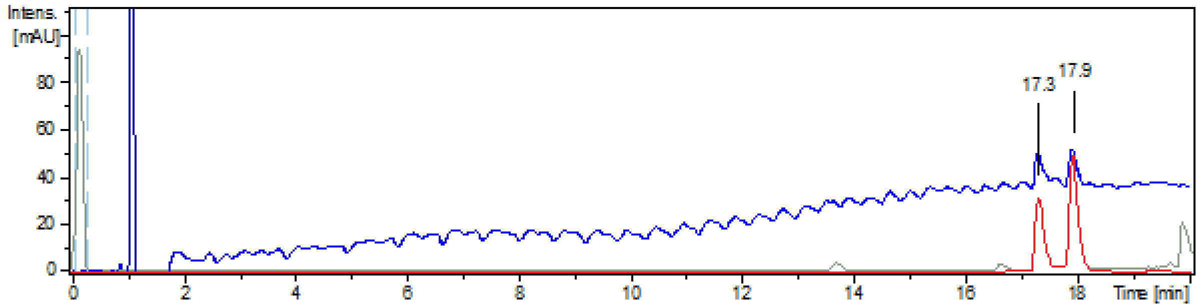

Supplementary Fig. 2 | HPLC-UV-MS chromatogram of **4** (Nucleoshell® EC RP-C18 column, 150 × 2 mm, 2.7 µm, flow rate: 0.3 mL min^{-1} with a linear gradient from 90:10 (0.1% FA in H_2O:0.06% FA in MeOH, *v*:*v*) to 0:100 (0.1% FA in H_2O:0.06% FA in MeOH, *v*:*v*) over 20 min. Monitored at λ = 220 nm. The BPC is shown in gray and the EIC of the *N*-methylated peptide **4** ($C_{47}H_{64}N_9O_6S$) with $[M+H]^+$, *m/z* 884.485 ± 0.005 for $C_{47}H_{65}N_9O_6S$ is shown in red.

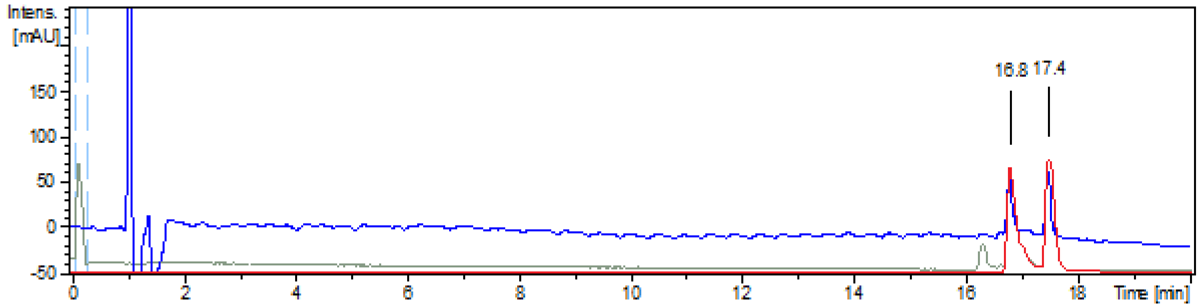

Supplementary Fig. 3 | HPLC-UV-MS chromatogram of **5** (Nucleoshell® EC RP-C18 column, 150 × 2 mm, 2.7 µm, flow rate: 0.3 mL min^{-1} with a linear gradient from 90:10 (0.1% FA in H_2O:0.06% FA in MeOH, *v*:*v*) to 0:100 (0.1% FA in H_2O:0.06% FA in MeOH, *v*:*v*) over 20 min. Monitored at λ = 220 nm. The BPC is shown in gray and the EIC of the *N*-methylated peptide **5** ($C_{47}H_{64}N_9O_6S$) with $[M+H]^+$, *m/z* 884.485 ± 0.005 for $C_{47}H_{65}N_9O_6S$ is shown in red.

Fragment ion analysis by LC-HRMS for *N*-methylated lugdunin analogues (3-5)

Fragmentation route molecules are shown in linearized form.

3-Methyl-3,6-ditrp-lugdunin (3). $[M+H]^+$ calculated for $C_{47}H_{65}N_9O_6S$, 884.4851; found 884.4852 (2.0 ppm err; 9.2 mSigma)

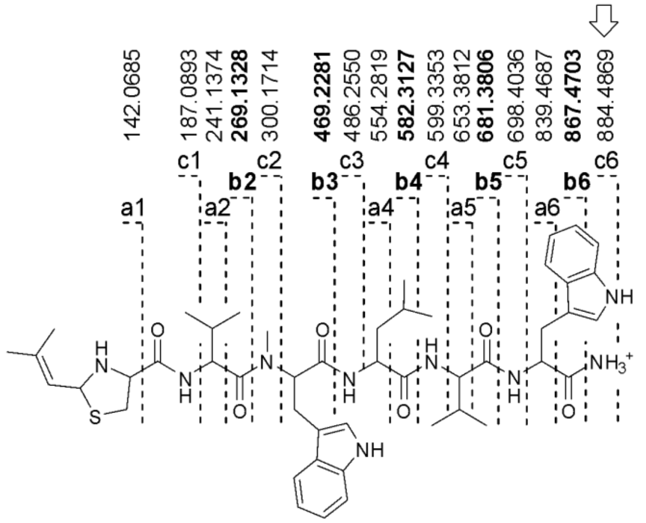

4-Methyl-3,6-ditrp-lugdunin (4). $[M+H]^+$ calculated for $C_{47}H_{65}N_9O_6S$, 884.4851; found 884.4852 (2.2 ppm err; 8.5 mSigma)

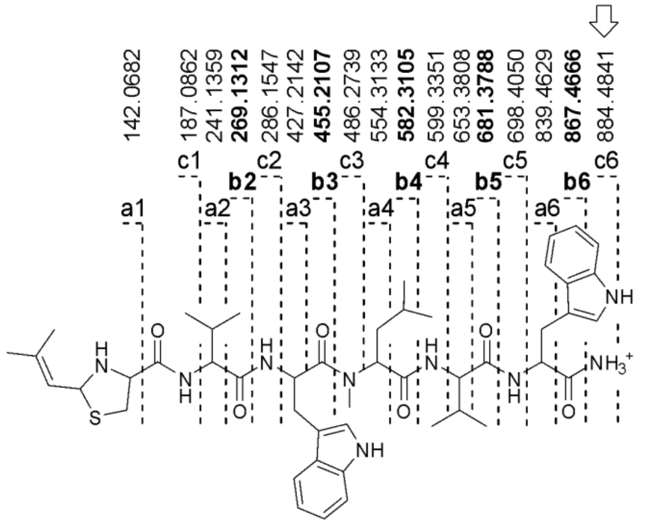

6-Methyl-3,6-ditrp-lugdunin (5). $[M+H]^+$ calculated for $C_{47}H_{65}N_9O_6S$, 884.4851; found 884.4852 (1.1 ppm err; 7.2 mSigma)

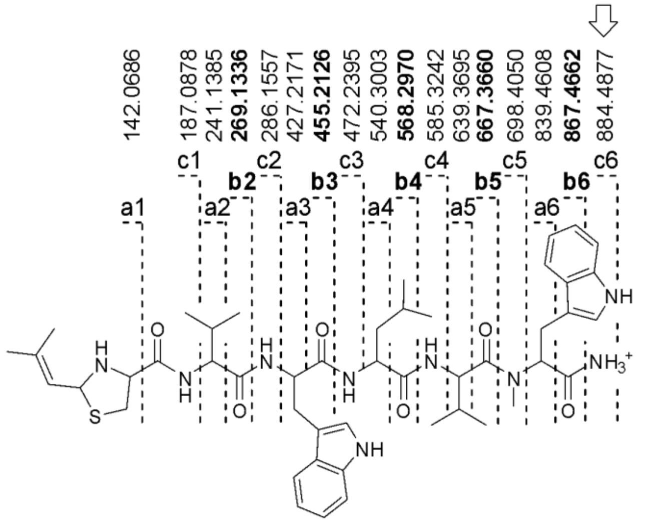

NMR spectra of *N*-methylated lugdunin analogues (3-5)

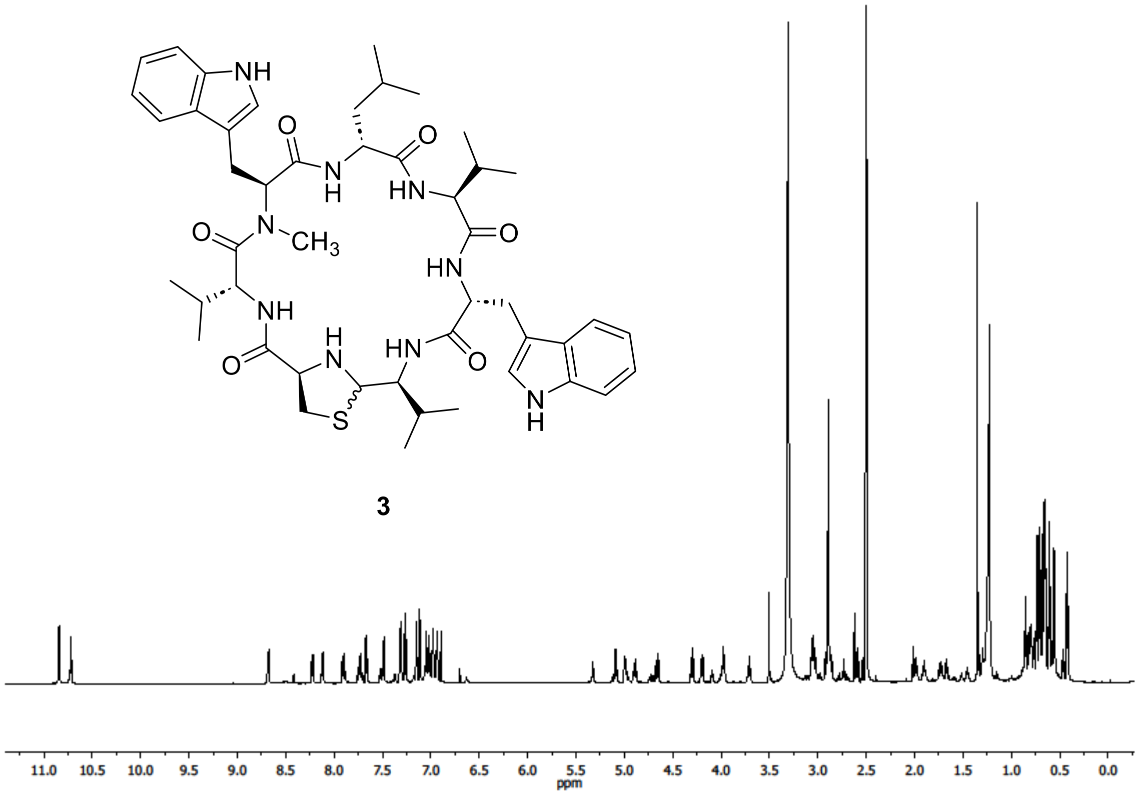

Supplementary Fig. 4 | ^{1}H-NMR spectrum of *N*-methylated lugdunin **3** (700 MHz, DMSO-d_6, 303 K).

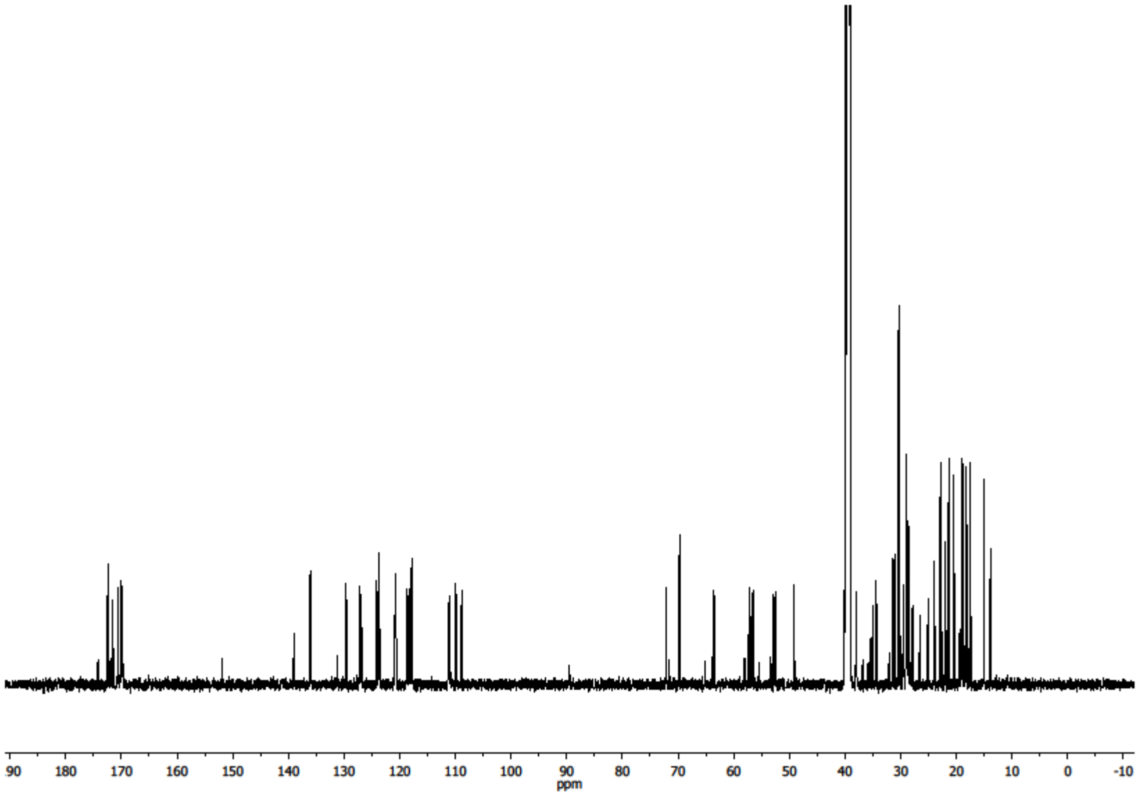

Supplementary Fig. 5 | ^{13}C-NMR spectrum of *N*-methylated lugdunin **3** (176 MHz, DMSO-d_6, 303 K).

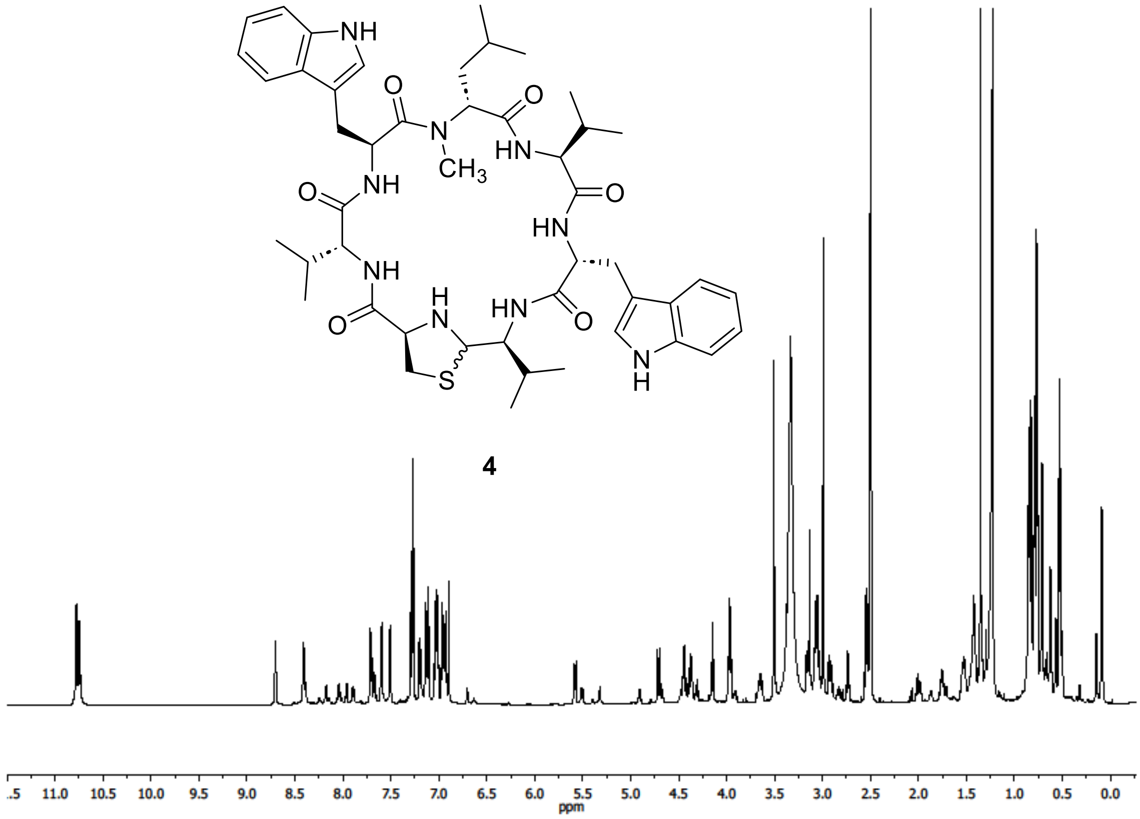

Supplementary Fig. 6 | ^{1}H-NMR spectrum of *N*-methylated lugdunin **4** (700 MHz, DMSO-d_6, 303 K).

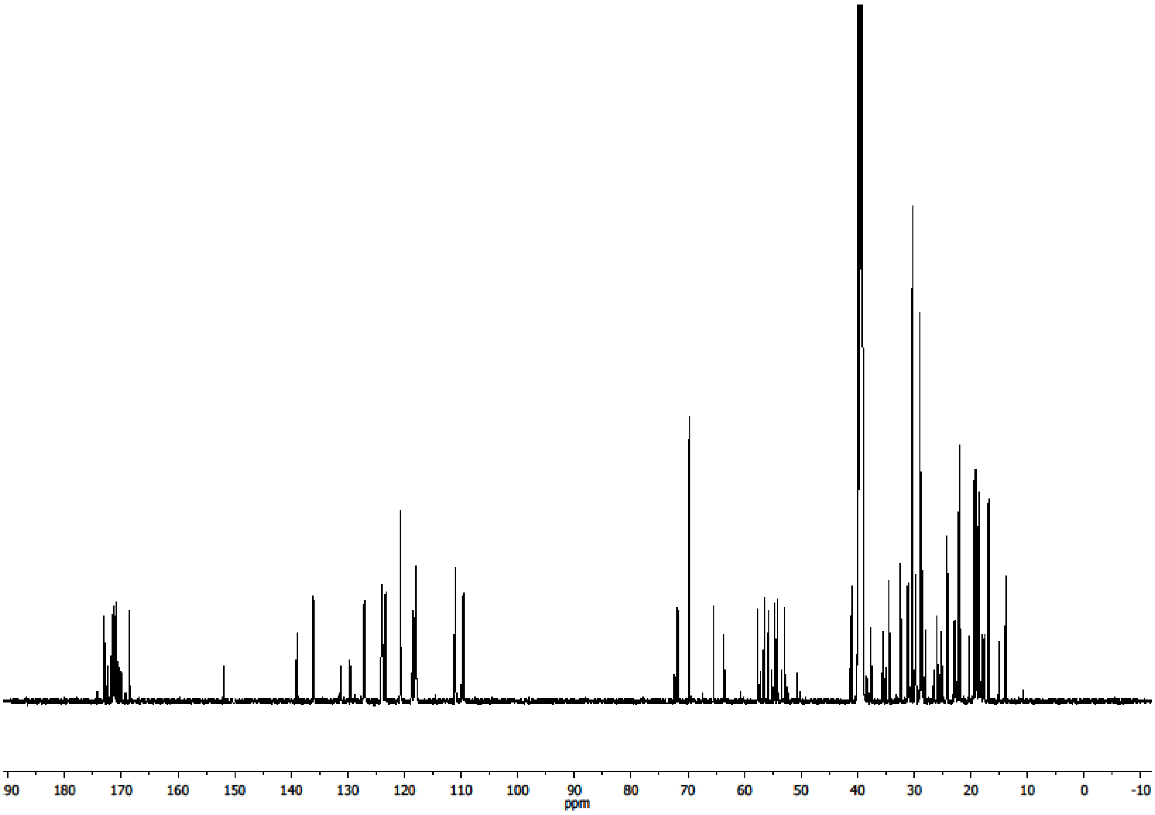

Supplementary Fig. 7 | ^{13}C-NMR spectrum of *N*-methylated lugdunin **4** (176 MHz, DMSO-d_6, 303 K).

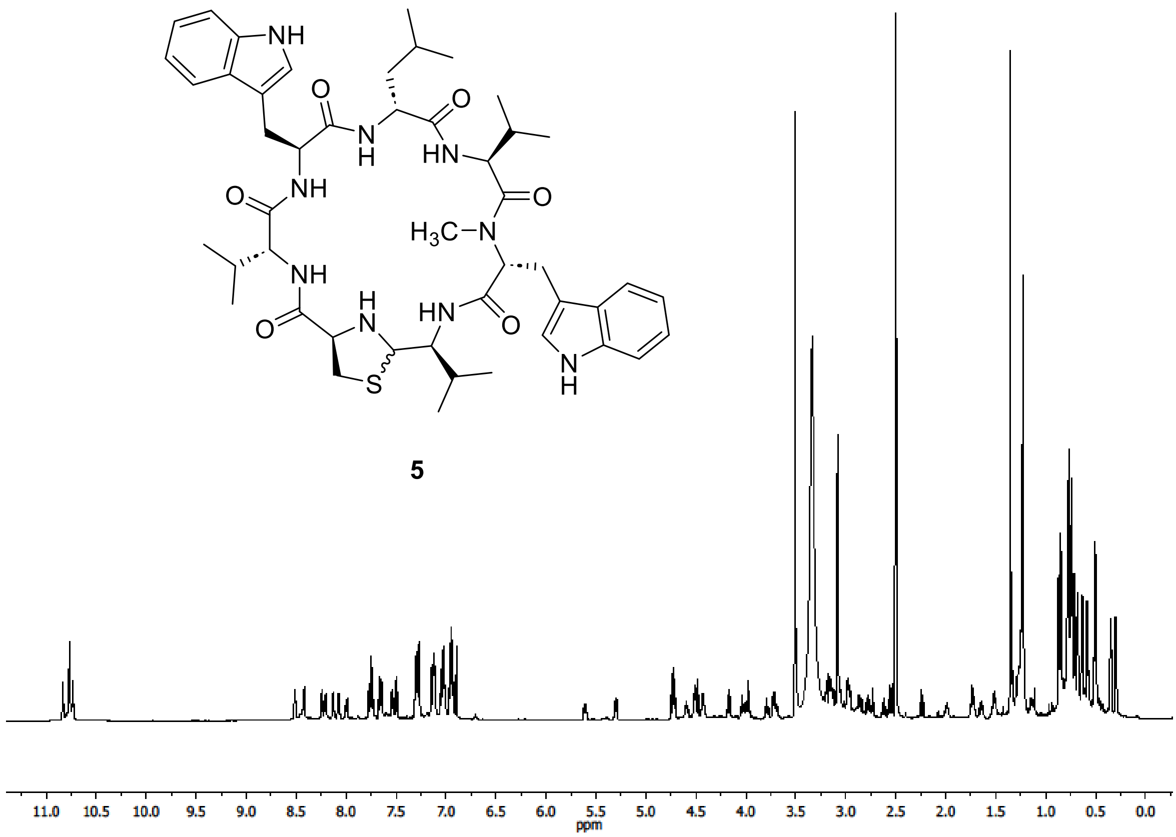

Supplementary Fig. 8 | ^{1}H-NMR spectrum of *N*-methylated lugdunin **5** (700 MHz, DMSO-d$_6$, 303 K).

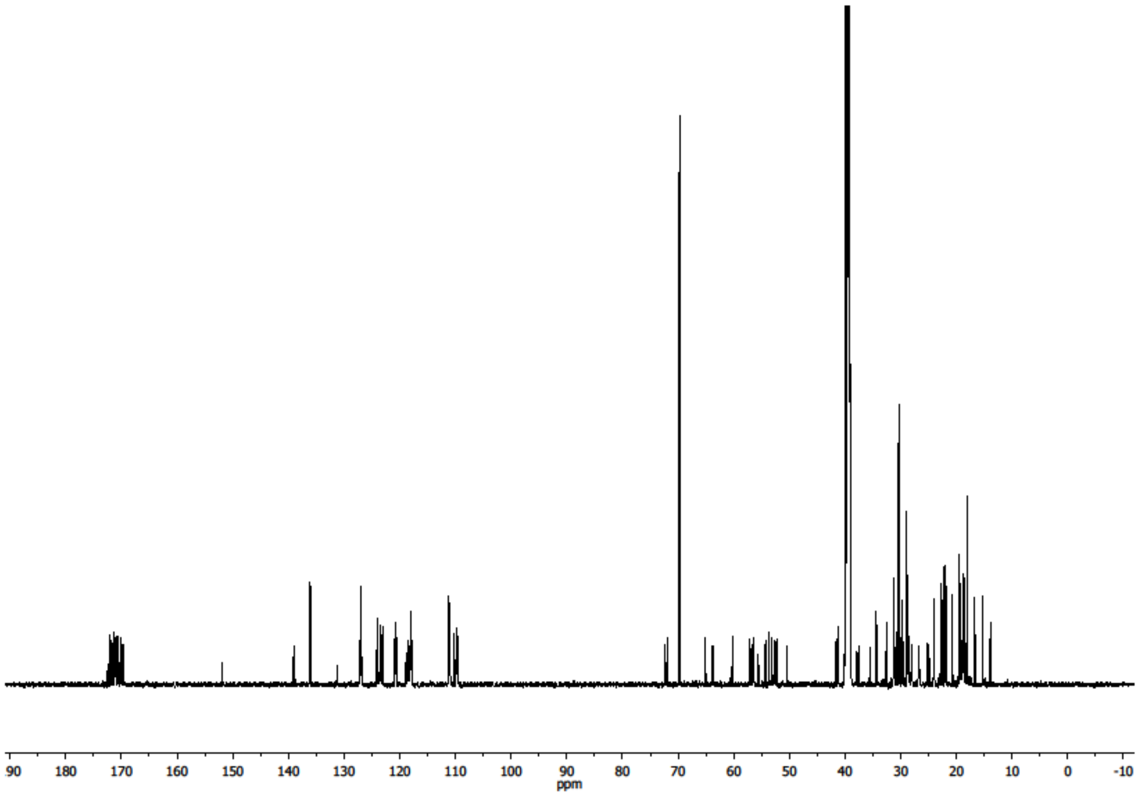

Supplementary Fig. 9 | ^{13}C-NMR spectrum of *N*-methylated lugdunin **5** (176 MHz, DMSO-d$_6$, 303 K).

Detailed experimental setup for orientational IR studies

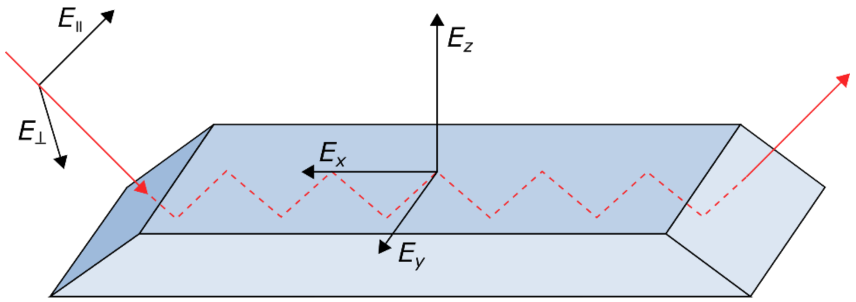

Supplementary Fig. 10 | Experimental setup for the determination of orientational properties.

Supplementary Table 1 | Parameters in the used ATR-setup to determine orientational parameters.

Parameter	Value
Refractive indices	n_1 = 4.00
	$n_2 = n_3$ = 1.43
Incident angle	α = 45°
Electric field amplitudes	E_x = 1.40
	E_y = 1.51
	E_z = 1.62
Transition dipole moment angle	$\alpha_{\text{Acyl chain}}$ = 90 °
	$\alpha_{\text{Peptide nanotube}}$ = 0°

Molecular dynamics simulations of lugdunin

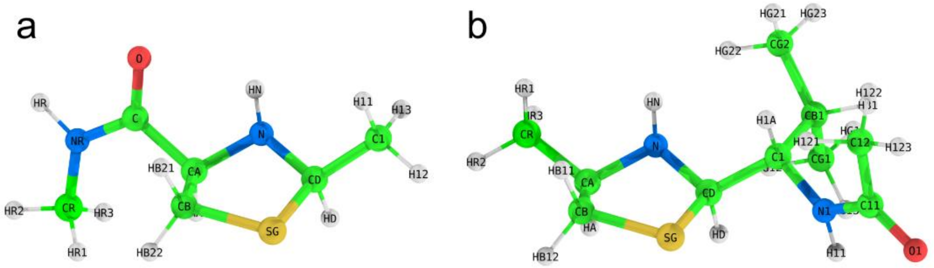

Supplementary Fig. 11 | Molecular structures of (**A**) molecule A and (**B**) molecule B, optimized with the MP2/6-31G(d) model chemistry.

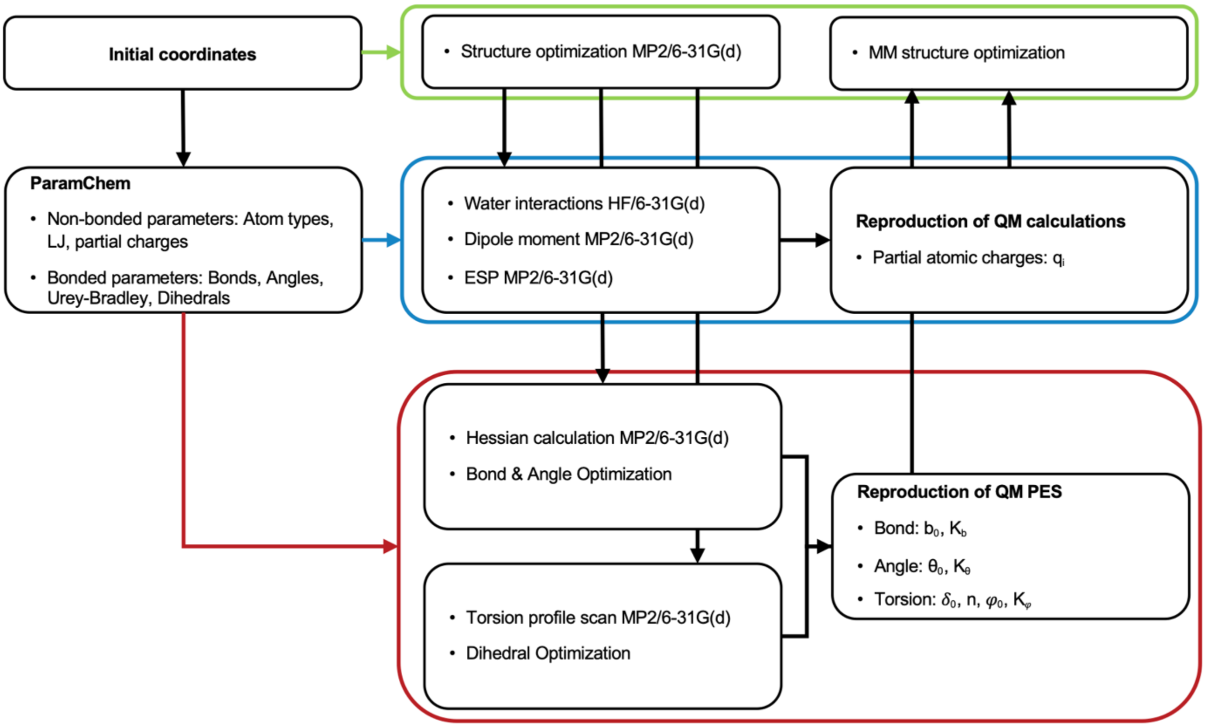

Supplementary Fig. 12 | Workflow for the parameterization process. Initial coordinates were used as input for the ParamChem webserver[1]. Initial coordinates and parameters were further refined during the structure optimization (green), the charge fitting (blue), and the bonded parameter optimization (red). The output of each step was used to refine the MM model and improve the accuracy of the final parameters.

Supplementary Table 2 | QM, optimized and initial interaction energies and distances for each orientation (1,2) of the probe sites in molecule A.

N	Probe site	Angle	Energy ($QM^1/Opt^1/Init^1$ $QM^2/Opt^2/Init^2$)	Distance ($QM^1/Opt^1/Init^1$ $QM^2/Opt^2/Init^2$)
1	HA	0°	-1.83/-2.42/-1.86 -2.10/-2.71/-2.50	2.96/2.82/2.90 2.76/2.67/2.69
2	HB22	0°	-1.64/-1.45/-1.36 -1.67/-1.82/-1.70	2.72/2.74/2.75 3.07/3.09/3.11
3	HD	0°	-1.21/-0.92/-1.22 -1.30/-1.08/-1.02	2.63/2.74/2.71 2.61/2.72/2.73
4	HN†	0°	-0.27/-0.73/ 0.87 -2.37/-2.63/-3.54	2.24/2.23/2.49 1.99/1.94/1.90
5	HN†	45°	0.82/-0.08/ 1.35 -1.93/-2.37/-3.26	2.69/2.33/2.78 2.03/1.95/1.91
6	HN†	90°	0.38/-0.42/ 1.15 -2.98/-2.90/-3.80	2.52/2.28/2.62 1.96/1.93/1.90
7	HN†	135°	-1.07/-1.15/ 0.58 -3.48/-3.19/-4.11	2.10/2.18/2.10 1.92/1.92/1.88
8	N	0°	-6.31/-6.62/-5.15 -6.38/-6.80/-6.28	1.91/1.91/1.98 1.91/1.90/1.92
9	N	90°	-5.36/-5.35/-3.95 -5.37/-5.26/-4.98	1.92/1.93/2.00 1.92/1.92/1.93
10	N	180°	-4.82/-4.69/-4.11 -5.57/-5.29/-5.10	1.94/1.95/1.99 1.93/1.93/1.94
11	N	270°	-6.96/-6.63/-5.84 -7.95/-7.63/-7.16	1.88/1.91/1.96 1.87/1.90/1.91
12	SG	0°	-1.62/-1.90/-0.73 -1.56/-1.96/-1.89	2.88/2.41/2.57 2.88/2.40/2.40
13	SG	90°	-1.94/-2.18/-1.03 -1.91/-2.22/-2.14	2.82/2.39/2.54 2.81/2.38/2.38
14	SG	180°	-1.72/-2.01/-0.92 -1.73/-2.00/-1.97	2.87/2.39/2.54 2.86/2.39/2.40
15	SG	270°	-1.79/-1.80/-0.65 -1.80/-1.81/-1.79	2.83/2.41/2.58 2.82/2.40/2.41

†Probe site was only used in the second optimization round.

Supplementary Table 3 | Metrics of the water interactions, the dipole moment, and the ESP before and after the charge fitting process for molecule A.

Iteration	Objective function (Opt/Init)	$RMSD^{Energy}$ (Opt/Init)	$RMSD^{Distance}$ (Opt/Init)	Angle (Opt/Init)	QM/MM Dipole (Opt/Init)	$RMSD^{ESP}$ (Opt/Init)
1	8.70/21.66	0.29/0.89	0.28/0.18	10.72/36.15	2.96, -1.51 1.95<br>2.72 -2.24 2.08	2.09/3.87
2	7.72/10.85	0.32/0.61	0.24/0.24	8.96/9.50	2.36, -2.14, 1.4<br>2.48, -3.17, 1.86	2.44/2.15

Supplementary Table 4 | Optimal and initial partial atomic charge of the atoms in the thiazolidine moiety.

Atom	Optimal	Initial
CA	0.274	0.227
CB	-0.216	-0.221
CD	0.182	0.161
N	-0.783	-0.748
HN	0.352	0.378
SG	-0.169	-0.157

Supplementary Table 5 | Statistics of the structural (GeomDelta) and energy (EnDelta) differences before and after the bonded parameter optimization process for molecule A.

Index	Atom type 1	Atom type 2	Atom type 3	GeomDelta (Opt¹/Init¹ Opt²/Init²)	EnDelta (Opt¹/Init¹ Opt²/Init²)
1	CG331	CG3C51	NG3C51	-1.00/-15.32 -0.01/ 5.44	-0.50/ 0.11 -0.46/-0.12
2	CG331	CG3C51	SG311	-1.05/ 6.47 -0.05/-3.48	0.05/ 0.11 0.16/-0.01
3	CG3C51	NG3C51	CG3C51	0.55/ -5.00 0.02/ 1.61	0.04/ 1.07 -0.08/-0.52
4	CG3C51	SG311	CG3C52	0.47/ -1.57 0.09/ 1.13	-0.11/-0.05 -0.16/ 0.01

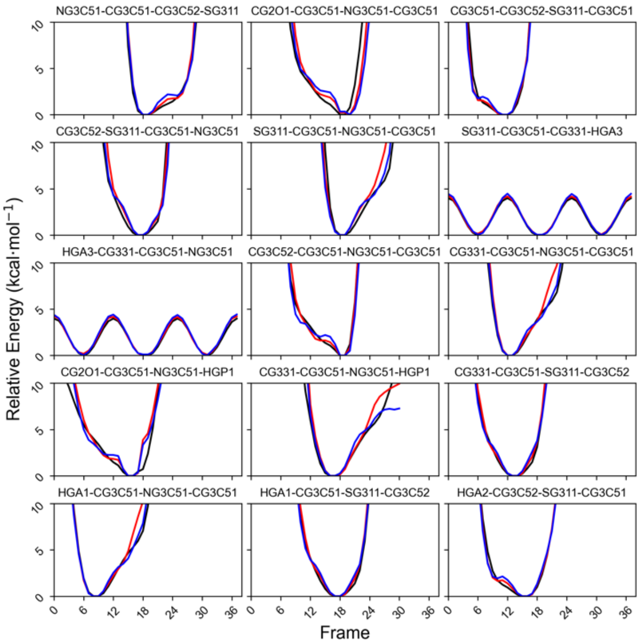

Supplementary Fig. 13 | QM torsion profiles (black) and optimized MM torsion profiles for the first (red) and second (blue) optimization iteration of molecule A.

Supplementary Table 6 | Statistics of the structural (GeomDelta) and energy (EnDelta) differences before and after the bonded parameter optimization process for molecule B.

**Index**	**Atom type 1**	**Atom type 2**	**Atom type 3**	**GeomDelta** (Opt¹/Init¹ Opt²/Init²)	**EnDelta** (Opt¹/Init¹ Opt²/Init²)
1	CT1	CG3C51	SG311	0.03/ 6.21 0.00/-0.59	0.07/0.21 0.03/ 0.02
2	CT1	CG3C51	NG3C51	0.00/-8.81 0.00/-0.87	-0.11/0.20 0.03/-0.08

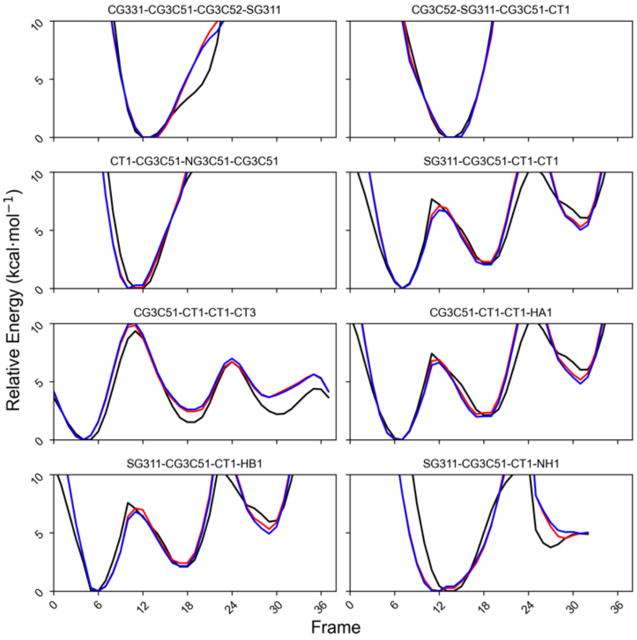

Supplementary Fig. 14 | QM torsion profiles (black) and optimized MM torsion profiles for the first (red) and second (blue) optimization iteration of molecule B.

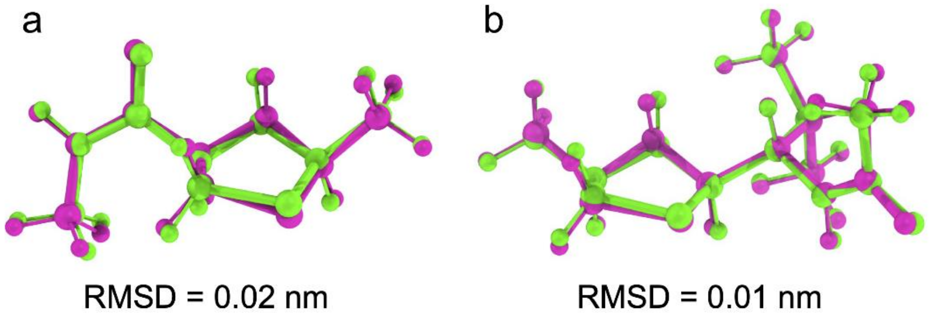

Supplementary Fig. 15 | RMSD of the aligned QM (green) and MM (magenta) optimized structure for (**A**) molecule A and (**B**) molecule B.

Supplementary Table 7 | Systems studied in all-atom molecular dynamics simulations. A model membrane resembling the cell membrane of a gram-positive bacterial strain (*Bacilius subtilis,* abbreviated as Gram+) was taken from an earlier simulation study by Pogozheva *et al.*[2]. Its phospholipid composition splits into 65 mol% PG, 27 mol% PE, and 8 mol% Cardiolipin, with most lipid tails being branched with iso/anteiso C15 fatty acids. Structures are listed in Supplementary Fig. 16. For the Gram+ model membrane, we sequentially added lugdunin molecules, using the equilibrated membrane at lower peptide-to-lipid ratios as starting model for the increased peptide concentration.

System	Variant	Replicas/ Windows	Peptide	Lipids	T / *K*	Time in *ms*	Box / nm^3	Water/Lipid
**Channel stability**								
DMPC	$Lugd^{WT}$	9	3	160	310.15	8x 2.78; 10	7 x 7 x 8	50.6
DMPC	$Lugd^{WT}$	9	4	160	310.15	8x 2.81; 10	7 x 7 x 8	50.6
DMPC	$Lugd^{WT}$	9	5	160	310.15	8x 1.26; 10	7 x 7 x 8	50.6
DOPC	$Lugd^{WT}$	9	4	160	298.15	8x 3.30; 10	7 x 7 x 8	50.6
DOPC (same)	$Lugd^{WT}$	9	4	160	298.15	8x 1.86; 10	7 x 7 x 8	50.6
DOPC	$Lugd^{WT}$	9	5	160	298.15	8x 3.13; 10	7 x 7 x 8	50.6
POPC	$Lugd^{WT}$	9	4	160	298.15	8x 2.66; 10	7 x 7 x 8	50.6
POPC	$Lugd^{WT}$	9	5	160	298.15	8x 2.67; 10	7 x 7 x 8	50.6
POPC/Chol	$Lugd^{WT}$	9	4	160	298.15	8x 3.28; 1.36	6 x 6 x 11	50.6
POPC/Chol (same)	$Lugd^{WT}$	9	4	160	298.15	8x 2.39; 10	6 x 6 x 11	50.6
POPC/Chol	$Lugd^{WT}$	9	5	160	298.15	8x 3.32; 10	6 x 6 x 11	50.6
**Umbrella Sampling**								
POPC	$Lugd^{WT}$	104	1	160	298.15	104x 1.0	7 x 7 x 11	76
POPC/Chol (20 mol%)	$Lugd^{WT}$	86	1	160	298.15	86x 1.0	6 x 6 x 11	59.7
POPC	$Lugd^{Cys4Ala}$	77	1	160	298.15	77x 1.0	7 x 7 x 11	76
POPC/Chol (20 mol%)	$Lugd^{Cys4Ala}$	79	1	160	298.15	79x 1.0	6 x 6 x 11	59.7
Gram+	$Lugd^{WT}$	78	1	160	298.15	78x 1.0	7 x 7 x 11	79.7
**Spontaneous insertion**								
POPC	$Lugd^{WT}$	4	4	160	298.15	4x 4.55	7 x 7 x 8.6	49.4
POPC/Chol	$Lugd^{WT}$	4	4	160	298.15	4x 4.7	6 x 6 x 11	49.1
Gram+	$Lugd^{WT}$	4	5; 10; 15; 20	200	298.15	2.75; 10; 1; 10	8 x 8 x 8.5	49.9; 48.4; 47.1; 45.6

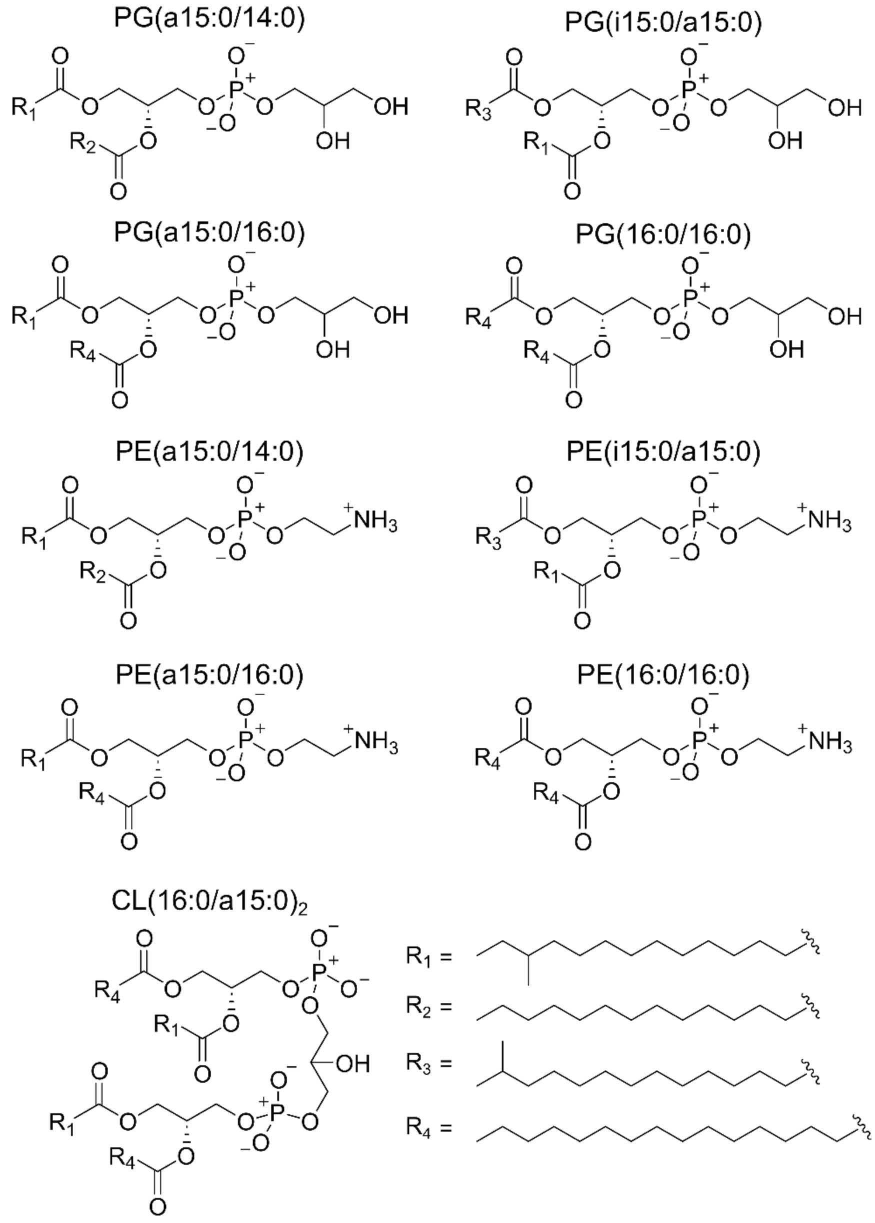

Supplementary Fig. 16 | Lipids included in the model for Gram-positive membranes. Composition adapted from Pogozheva *et al.*[2]: 22 mol% PG(a15:0/14:0), 27 mol% PG(i15:0/a15:0), 9 mol% PG(a15:0/16:0), 7 mol% PG(16:0/16:0), 9 mol% PE(a15:0/14:0), 11 mol% PE(i15:0/a15:0), 4 mol% PE(a15:0/16:0), 3 mol% PE(16:0/16:0), 8 mol% CL(16:0/a15:0).

Partitioning of lugdunin into different lipid compositions

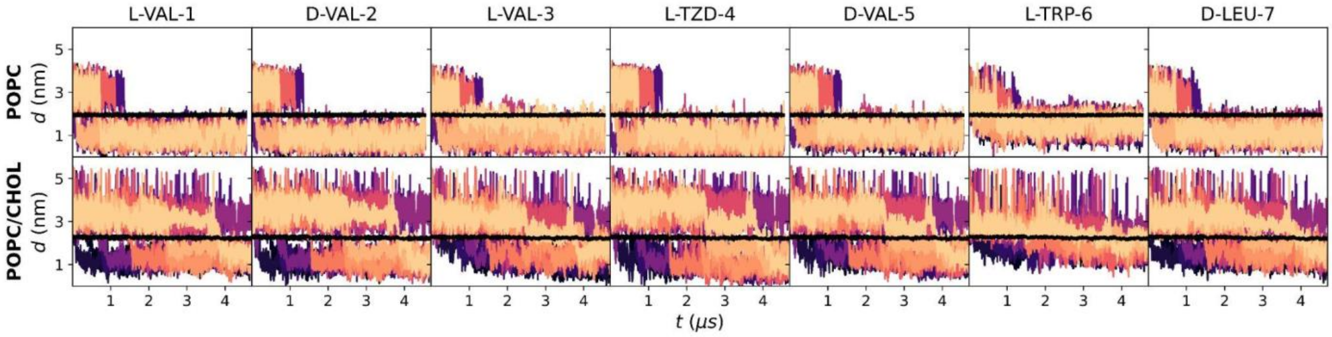

Supplementary Fig. 17a | Spontaneous insertion of lugdunin into phospholipid bilayers as observed in all-atom molecular dynamics simulations. Distance of lugdunin amino acids (nomination as in Scheme 1) from the bilayer core as a function of simulation time for pure POPC (top row) and POPC/cholesterol (7:3) (bottom row). Data were obtained from four replicas MD simulations with four lugdunin molecules each (different colours). Each simulation started with lugdunin randomly inserted into the water phase. The black line shows the normal position of the centre of mass of the lipid phosphate groups.

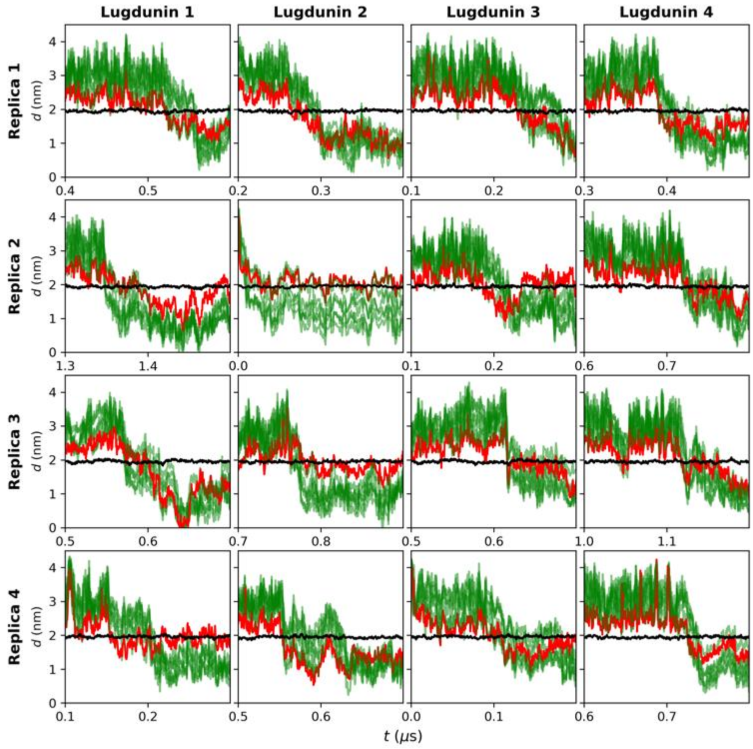

Supplementary Fig. 17b | Spontaneous insertion of lugdunin into pure POPC bilayers. Shown are the distances of lugdunin amino acids (L-TRP-6 in red, others in green) from the bilayer core, ≈ 100 ns before and after the insertion into the membrane. The initial contact of lugdunin with the membrane is made by tryptophan, followed by the insertion of all other side chains into the membrane. Each simulation started with four lugdunin molecules randomly inserted into the water phase. The black line shows the normal position of the centre of mass of the lipid phosphate groups.

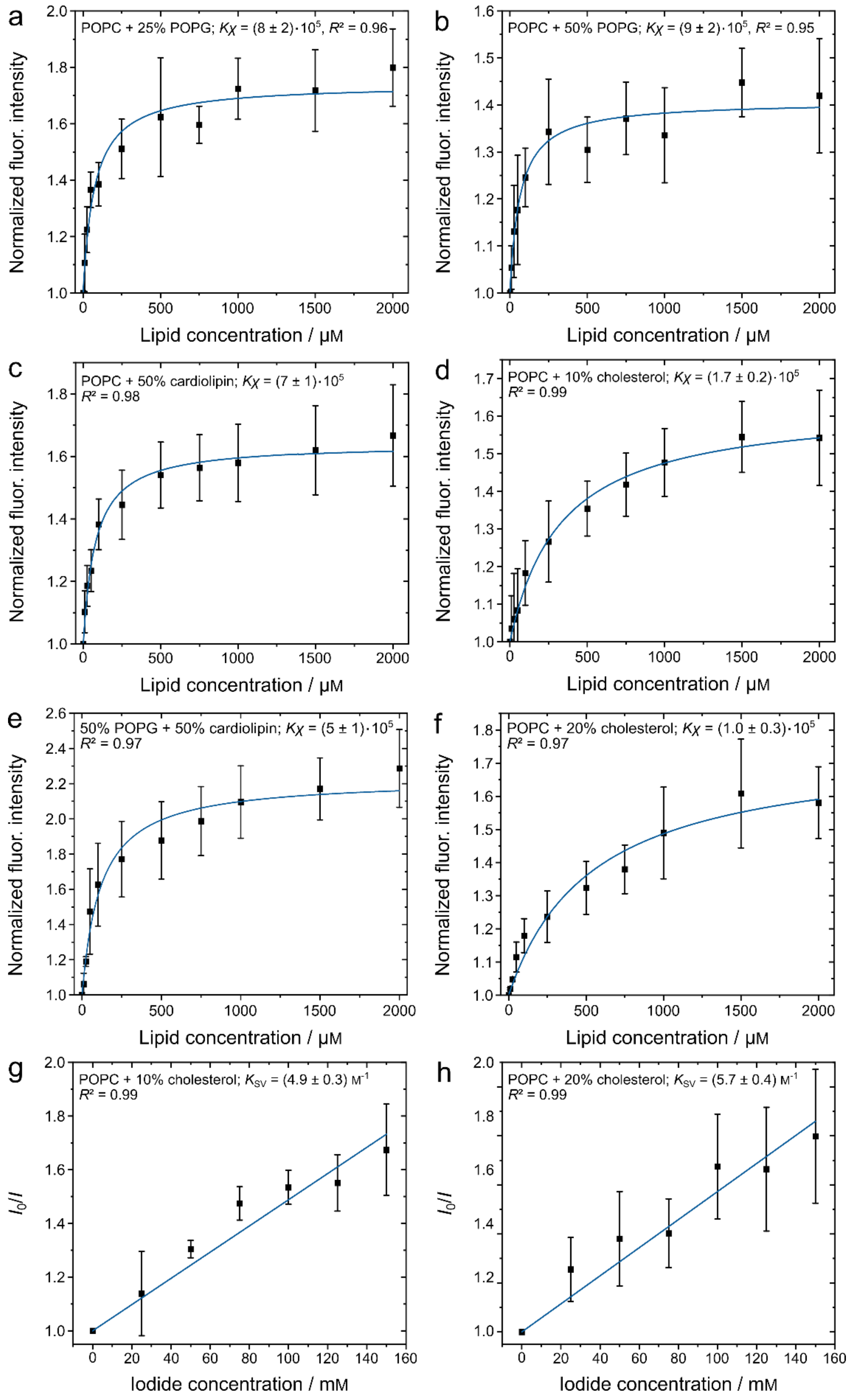

Supplementary Fig. 18 | Mean partition coefficients and Stern-Volmer plots for different lipid compositions (*n* ≥ 3 independent experiments). The error bars are the standard deviation of the mean.

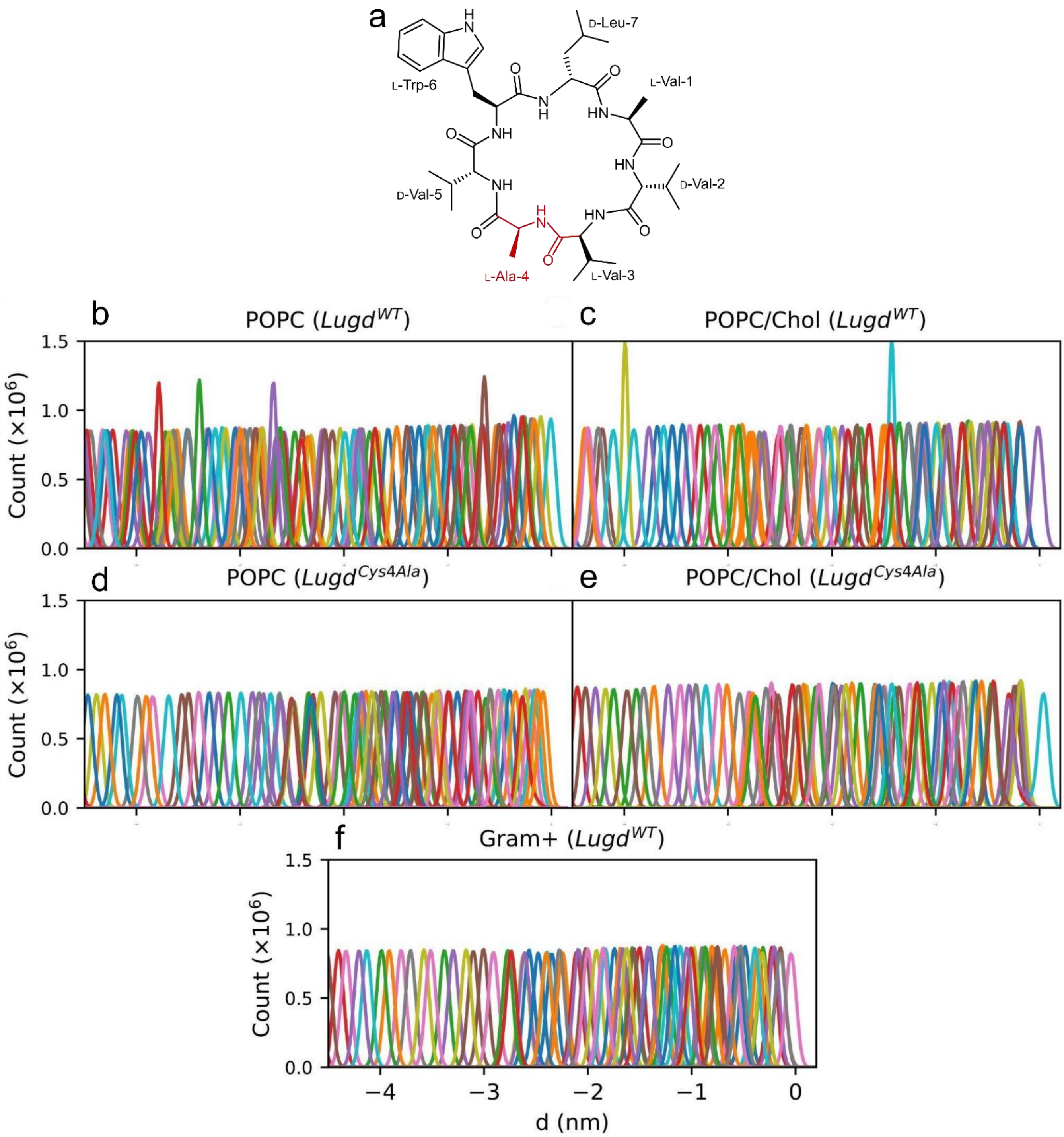

Supplementary Fig. 19 | Histograms of umbrella simulations used in the calculation of the potential of mean force (PMF) profiles for the insertion of lugdunin and a lugdunin variant (thiazolidine replaced by alanine) into differently composed model membranes.

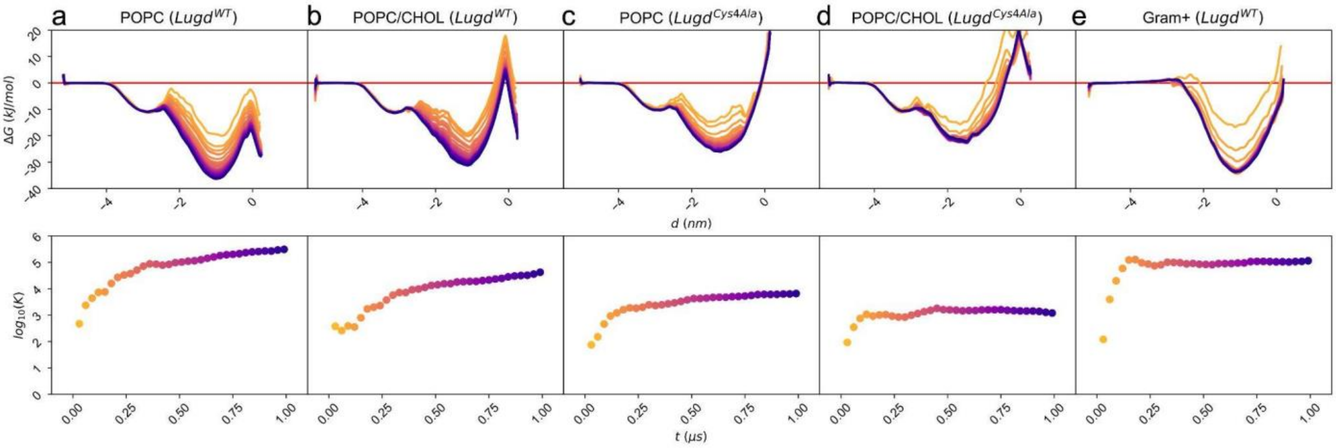

Supplementary Fig. 20 | Convergence of the potential of mean force (PMF) for lugdunin insertion into different model membranes as a function of simulation time for each umbrella system.

Molecular orientation of lipid multi-bilayers

To choose an appropriate lipid matrix for the investigation of structural and orientational properties of lugdunin, we evaluated the molecular order of lipid multi-bilayer systems composed of either POPC, DPPC or DMPC. The resulting values for the dichroic ratio, the order parameter and the effective tilt angle with respect to the IRE normal indicate that DPPC and DMPC form much more ordered multi-bilayers compared to POPC (Supplementary Table 8). As DMPC has a lower main phase transition temperature than DPPC and forms fluid membranes at room temperature, we employed this lipid for further IR-spectroscopic investigations.

Supplementary Table 8 | Dichroic ratio, order parameter and effective tilt angle of pure lipid multi-bilayers composed of POPC, DPPC or DMPC ($n \geq 2$ experiments, mean ± standard deviation).

**Lipid**	R^{ATR}	$S(\Theta)$	Θ / °
POPC	1.56 ± 0.02	0.29 ± 0.02	43.4 ± 0.5
DPPC	1.12 ± 0.02	0.68 ± 0.02	27.4 ± 0.6
DMPC	1.12 ± 0.01	0.69 ± 0.01	27.2 ± 0.5

IR spectra of lugdunin for different peptide-to-lipid ratios

To discern the influence of the peptide concentration, we recorded IR spectra of lugdunin located in DMPC multi-bilayers at varying peptide-to-lipid ratios. For peptide-to-lipid ratios smaller than 1:20, the spectra displayed solely an antiparallel β-sheet structure. Larger peptide-to-lipid ratios revealed the emergence of a peak located at ~1624 cm^{-1} which we attribute to an aggregation of peptide monomers induced at high concentrations.

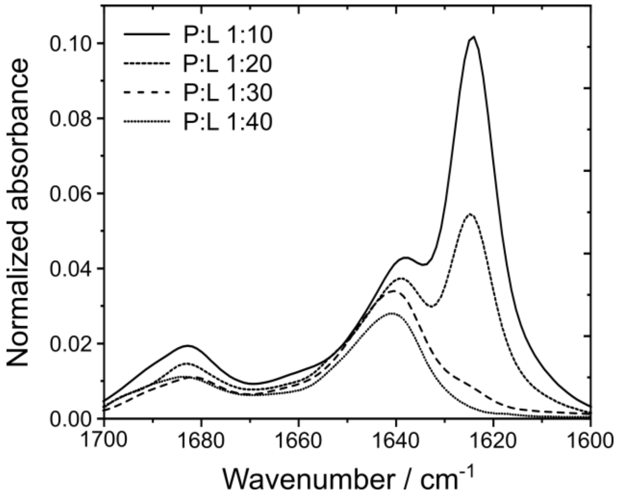

Supplementary Fig. 21 | ATR-FTIR spectra of lugdunin at different nominal peptide-to-lipid ratios.

Orientational parameters of lugdunin in lipid multi-bilayers

Supplementary Table 9 | Dichroic ratio R^{ATR}, order parameter *S*(Θ) and effective tilt angle Θ of DMPC multi-bilayers without and with lugdunin and a peptide-to-lipid ratio of 1:40 (*n*/*n*).

	DMPC[a]				DMPC + lugdunin[a]			
	$\tilde{\nu}$ / cm^{-1}	R^{ATR}	*S*(Θ)	Θ / °	$\tilde{\nu}$ / cm^{-1}	R^{ATR}	*S*(Θ)	Θ / °
CH_2	2919, 2851	1.13 ± 0.01	0.68 ± 0.01	27.5 ± 0.6	2919, 2851	1.28 ± 0.01	0.53 ± 0.02	34.0 ± 0.6[b]
Amide I	-	-	-	-	1641	3.3 ± 0.2	0.28 ± 0.03	44 ± 2[b,c]

[a] Data are mean ± standard deviation of 2-3 independent samples. [b] Tilt angles are relative to the surface of the internal reflection element and were calculated from the transition dipole moment of the respective vibrational mode. [c] The tilt angle relative to the membrane normal was determined to (10 ± 2)°.

Lucigenin quenching assay

To discharge that lugdunin does not transport HCl across lipid membranes leading to similar observations in the pyranine-based proton assay, we entrapped lucigenin in lipid vesicles exposed to a chloride gradient. The addition of lugdunin did not influence the resulting time courses demonstrating that the peptide does not mediate the translocation of chloride ions.

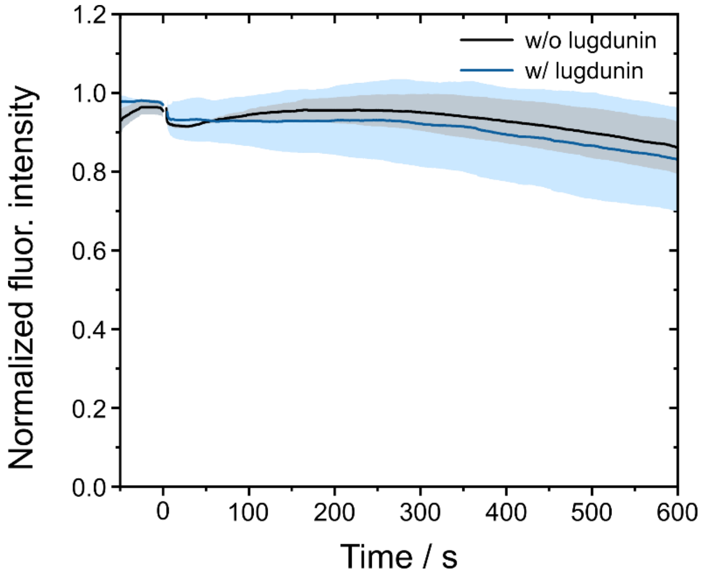

Supplementary Fig. 22 | Fluorescence time course of lucigenin entrapped in lipid vesicles exposed to a chloride gradient of 10 mM in the absence or the presence of lugdunin (peptide-to-lipid ratio 1:250, *n*/*n*). The shaded area represents the standard deviation of the mean from n = 3 experiments.

Setup and analysis of molecular dynamics simulations of channels composed of lugdunin peptides

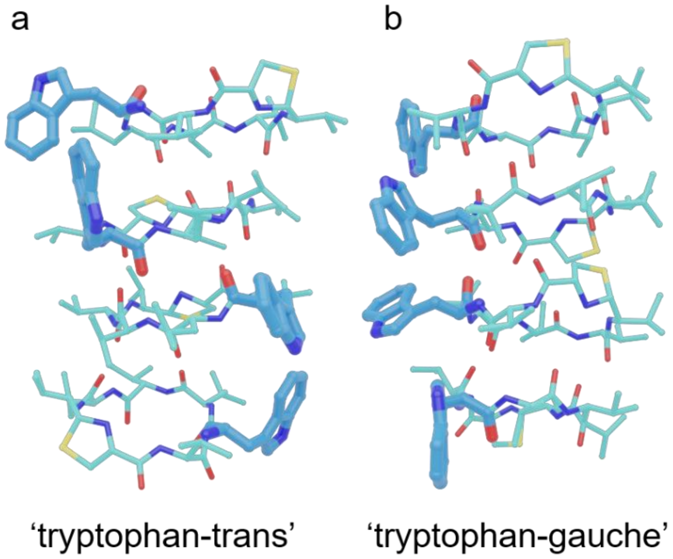

Supplementary Fig. 23 | Tryptophan-trans and gauche configurations for channels composed of four lugdunin peptides. These conformers were embedded in different lipid membranes and their stability was addressed in microsecond all-atom molecular dynamics simulations. Images were generated with Blender[2] and Molecular Nodes[3].

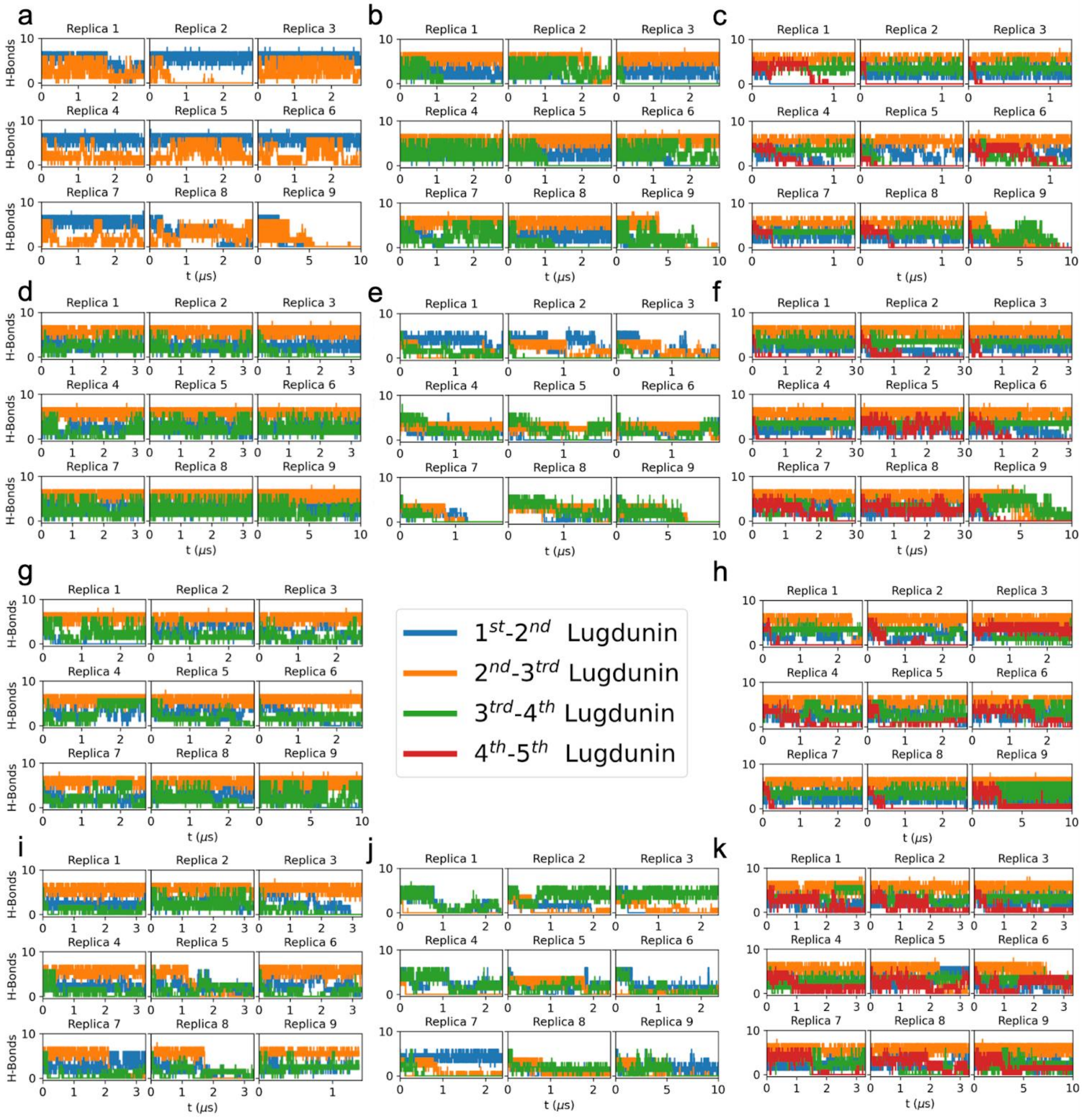

Supplementary Fig. 24 | Stability of channels composed of 3-5 lugdunin peptides in two different configurations (see Fig. 23, trans: a, b, c, d, f, g, h, i, k; gauche: e, j). Hydrogen bond network in lugdunin stacks over time in different lipid membranes: (a-c) DMPC, (d-f) DOPC, (g-h) POPC, and (i-k) POPC/Chol (70:30). Interactions were defined as pairs with acceptor-donor distances of 0.4 nm or less and acceptor-donor-hydrogen angles of 30° or less.

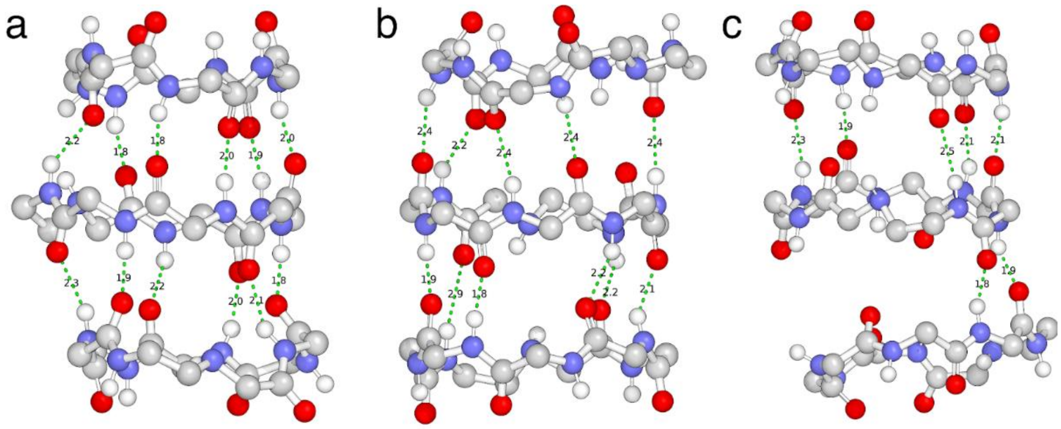

Supplementary Fig. 25 | Comparative snapshots of the hydrogen bond network in a trimeric lugdunin stack in a DMPC bilayer after **a** 0 μs, **b** 1.42 μs, and **c** 2.77 μs.

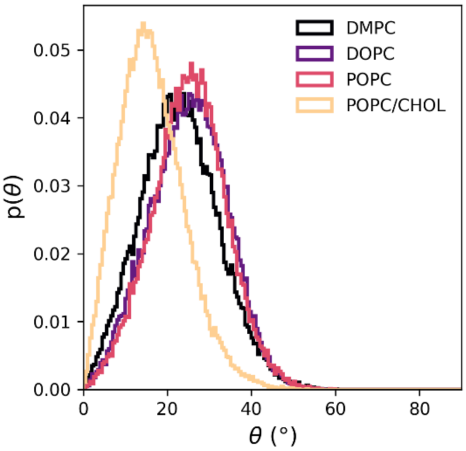

Supplementary Fig. 26 | Angle distribution of lugdunin channels with respect to the membrane normal. Analysed were the most stable configurations, i.e., lugdunin trimers in DMPC, tetramers in POPC and DOPC, and pentamers in POPC/Chol (70:30).

Single-channel properties of lugdunin nanotubes

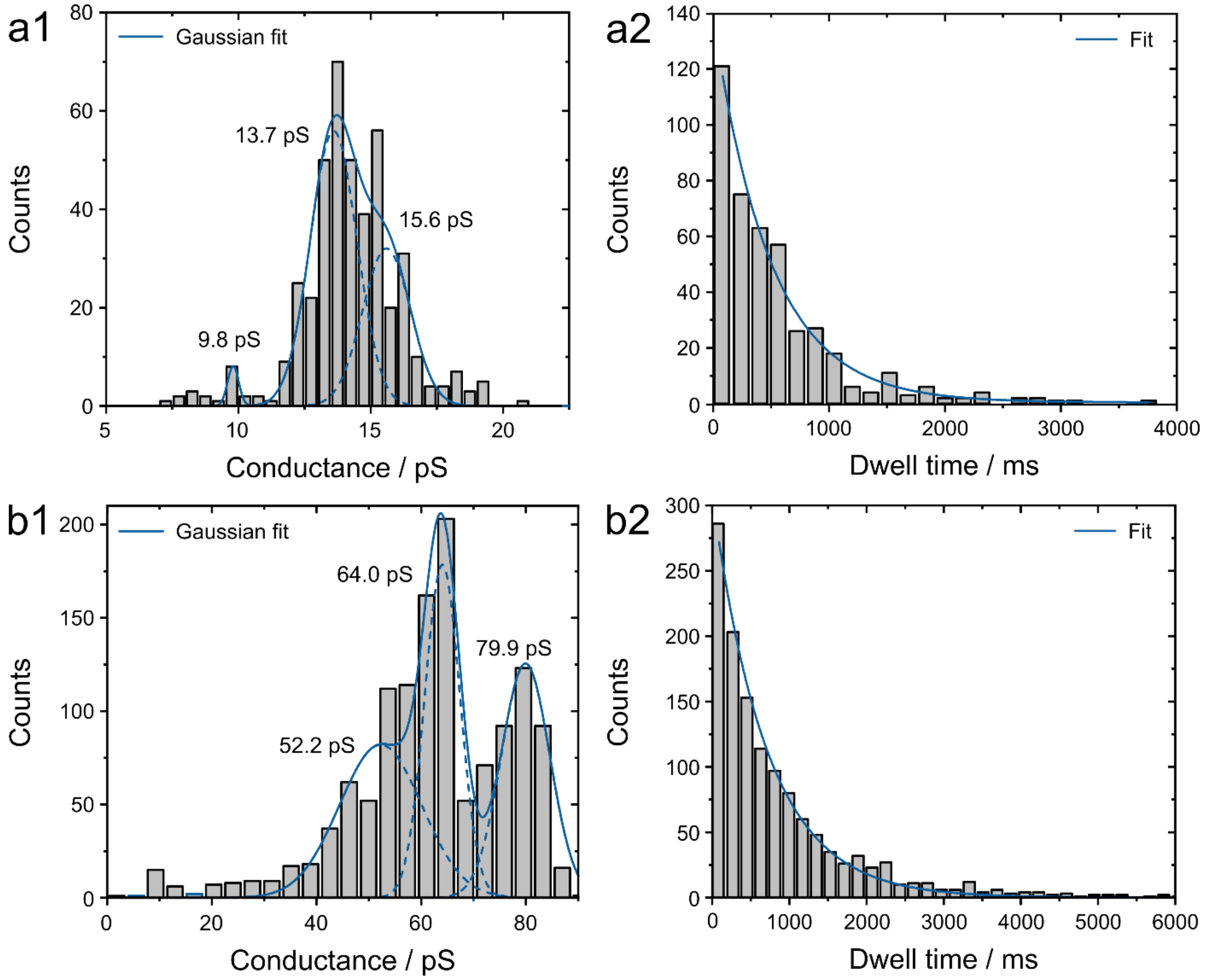

Supplementary Fig. 27 | Single-channel conductance event histograms recorded in 500 mM NaCl (a1, *n* = 432 events, bin width = 0.5 pS) or CsCl (b1, *n* = 1282 events, bin width = 3.7 pS) display three distinct conductance states for each tested alkali cation (Gaussian fits, blue). corresponding dwell time histograms yielding dwell time constants of (493 ± 34) ms for Na^+ (a2) and (705 ± 20) ms for Cs^+ (b2).

Supplementary Movies

Rendering of Supplementary Movie 1 and Supplementary Movie 2 was done with Blender v3.6[3] and Molecular Nodes v2.7.4[4].

Supplementary References

1. Vanommeslaeghe, K. & Mackerell, A. D. ParamChem webserver. Available at https://cgenff.silcsbio.com (2023).
2. Pogozheva *et al.*, Comparative molecular dynamics simulation studies of realistic eukaryotic, prokaryotic, and archaeal membranes. *J. Chem. Inf. Model.* **62**, 1036-1051 (2022).
3. Community, B. O. (2018), Blender - a 3D modelling and rendering package. Stichting Blender Foundation, Amsterdam. Retrieved from http://www.blender.org.
4. Brady Johnston, Yinying Yao, wjm41, Johannes Elferich, Olivier Laprevote, Thibault Tubiana, Domenico Marson, Jessica A. Nash, & Joyce. (2023). BradyAJohnston/MolecularNodes: v2.7.4 for Blender 3.5+ (v2.7.4). Zenodo.
