# Supplementary material for: The antimicrobial fibupeptide lugdunin forms water-filled channel structures in lipid membranes
Source: Nat Commun. 2024 Apr 25;15:3521. doi: 10.1038/s41467-024-47803-6 (PMC11045845; doi:10.1038/s41467-024-47803-6)
Supplement: Supplementary file 3 — Description of Additional Supplementary Files [file 41467_2024_47803_MOESM3_ESM.pdf]

Description of Additional Supplementary Files

File Name: Supplementary Movie 1
Description: Self-assembly of a trimeric lugdunin stack in a model membrane for a gram-positive bacterial strain[1]. The membrane surface is represented by the phosphorus atoms (orange spheres) of the phospholipids. Lugdunin molecules not part of the stack, as well as the remaining atoms of the lipid bilayer, hydrogen atoms, and solvent molecules are not shown. The movie displays a time span of 2.5 ms with a timestep of 3.5 ns, filtering out high-frequency motions. Rendering of the movie was done with Blender v3.6 and Molecular Nodes v2.7.4.

File Name: Supplementary Movie 2
Description: A self-assembled trimeric lugdunin stack in a model membrane for a gram-positive bacterial strain. Pink spheres represent sodium ions to counterbalance the charge of the phospholipid molecules. The stack works as a channel to transfer water (oxygen atoms displayed as red and hydrogen atoms as white spheres) across the membrane. Lipids, as well as hydrogen atoms of lugdunin are not shown. The movie displays a time span of 30 ns with a timestep of 50 ps. Rendering of the movie was done with Blender v3.6 and Molecular Nodes v2.7.4.
